# *Hsa-miR-27a-3p* overexpression in men with nonobstructive azoospermia: A case-control study

**DOI:** 10.18502/ijrm.v13i11.7963

**Published:** 2020-11-22

**Authors:** Hamid Norioun, Majid Motovali-bashi, Seyed Morteza Javadirad

**Affiliations:** Department of Cell and Molecular Biology and Microbiology, Faculty of Biological Science and Technology, University of Isfahan, Isfahan, Iran.

**Keywords:** hsa-miR-27a-3p, Male infertility, KDM3A.

## Abstract

**Background:**

The role of *KDM3A* and its downstream genes in male fertility has been approved in animal models. Additionally, the expression shrinkage of *KDM3A* is significantly correlated with human azoospermia phenotype. Aberrant expression of micro-RNAs could mislead spermatogenesis and mostly lead to diverse phenotypes of male infertility.

**Objective:**

The aim of this study was to evaluate the expression level of *hsa-miR-27a-3p* in azoospermic men to reveal its possible association with infertility.

**Materials and Methods:**

This case-control study was conducted on 30 azoospermic men, of whom, 19 had non obstructive azoospermia (NOA) and 11 obstructive azoospermia (OA) according to the pathological examinations. Comprehensive bioinformatics investigations were performed securely and *hsa-miR-27a-3p* was selected afterward. Reverse Transcriptase-quantitative polymerase chain reaction (RT-qPCR) method was used and statistical analysis was performed to compare the expression level of *hsa-miR-27a-3p* in both OA and NOA individuals.

**Results:**

In silico analysis suggested *hsa-miR-27a-3p*, with its potential binding ability to target *KDM3A* transcripts. The expression analysis of candidate *hsa-miR-27a-3p* indicated its significant overexpression in NOA men.

**Conclusion:**

The *hsa-miR-27a-3p* was overexpressed in NOA men compared to OA-control individuals. As a consequence, the overexpressed micro-RNA could downregulate directly *KDM3A *and indirectly *TNP1* and *PRM1*. Therefore, spermatogenesis could be misled and male infertility could be developed.

## 1. Introduction

Infertility and frequent abortion in couples are global concerns (1). Based on the world health organization guidelines, infertility is defined as the lack of ability to conceive after one year of intercourse without contraception (2). It has been reported that one-fourth of infertility cases are men-related and azoospermia is considered as one of the causes of male infertility (3). Azoospermia, the lack of sperm in an ejaculation, could be clinically nonobstructive (NOA) or obstructive (OA) with different etiologies and treatments (4). Unlike OA phenotype that shows normal spermatogenesis, the spermatogenesis process in NOA men is disturbed due to the exposure to toxic materials or abnormal evolution of the testes (5).

Disruption of epigenetic modification by a specific H3K9 demethylase (named Lysine-specific Demethylase 3A: *KDM3A*) causes post-meiosis deficiency of spermatid chromatin, sperm cells abnormal structure, and abnormal sperm shape (6, 7). The *KDM3a* knockdown mice studies confirmed that the expression of the gene starts at the late pachytene, continues till the late stage of spermatid creation, and that induces major effects on sperm maturation (6). The *KDM3A* function is extremely important not only for histone 3 demethylation but also for the removal of methyl groups from the promoter of downstream genes transition protein 1 (*TNP1*) and protamine 1 (*PRM1*) (6, 7). Without the function of *KDM3A* in round spermatids, excessive methylation would lead to the transcription suppression of *TNP1*/*PRM1* genes and the occurrence of infertility in animal models (8). These findings propose the essential role of *KDM3A* in the final development of male germline cells.

During the evolution of germline cells, the epigenetic modifications via non-coding RNAs (ncRNAs) are critical to regulate gene expression, genome stability, and genome imprinting (9). Micro-RNAs (miRNAs), an important class of ncRNAs with 18-25 nucleotide in length, are great regulators of gene expression (10). Several investigations have been conducted on human abnormally and their association with miRNAs (11, 12). Regarding the important roles of miRNAs in control of gene expression, this study investigates the effect of *hsa-miR-27a-3p* on *KDM3A* transcription and the ability of this miRNA as a diagnostic biomarker for azoospermia men.

## 2. Materials and Methods

In this case-control study, several biological databases such as:

• miRBase (http://www.mirbase.org),

• TargetScan (http://www.targetscan.org),

• DianamicroT (http://diana.imis.athenainnovation),

• miRanda (http://www.microrna.org),

• mirwalk (http://zmf.umm.uni-heidelberg.de),

• MirDB (http://www.mirdb.org),

• PicTar (https://pictar.mdc-berlin.de) and

• MiRGen (http://carolina.imis.athenainnovation.gr)

Were applied. Based on the interpretation of in silico studies, *hsa-miR-27a-3p* was selected as the best candidate against *KDM3A* transcripts. Based on the frequency of azoospermia in the population (1%) and in accordance with the previous studies, the minimum sample size of 30 was calculated (13). The sample size was obtained based on the following formula: 

$$\frac{{\left(Z_{1-a\setminus 2}\right)}^{2}p\left(1-p\right)}{d^{2}},$$

According to the histology clarification, out of 30 TESE and micro-TESE azoospermic sample tissues, 11 were OA and 19 were NOA. The average age of the individuals was 35 ± 5 yr with no background of testicular tumor or testis-related diseases. According to the previous studies, OA individuals were considered as our control group (14, 15).

Testicular tissue was collected from volunteer azoospermia men who referred for TESE and micro-TESE to the Isfahan Fertility and Infertility Center (IFIC) during a period of 2 yr from the spring 2013 to the spring 2015. Approximately 50 ml of testicular tissue was submerged in RNAlater stabilization solution (Ambion Life Science, Austin, TE, USA, AM7024). The proper amount of solution was chosen based on the manufacturer's instruction. Testicular tissues were stored at 4°C for 24 hr and homogenized using Heidolph Homogenizer DIAX 900 (Heidolph Instruments GmbH & CO. KG, Germany). Additionally, to eliminate cross-contamination, homogenizer probe was washed with 0.4 molar NaOH first, and then with ethanol 75%, and finally rinsed with distilled water. The total RNA was extracted using the SinaClon RNX_Plus extraction buffer (Sinaclon, IRAN) according to the manufacturer's instruction. The RNA quality and quantity were analyzed with 1% agarose gel electrophoresis and nanodrop OneC (Thermo Scientific, USA), respectively. Then 1 μl of the sample was loaded on the Nanodrop and the quantity of the extracted RNA was measured. RT-BON (Sinaclon, IRAN) adaptor primer was used to add a poly (A) tail to the end of extracted miRNAs and first-strand cDNA synthesis was done using oligo (dT) primers. High specific forward and reverse primers of *hsa-miR-27a-3p* were purchased from the Bonyakhteh Co. (Iran, http://strc.ac.ir/en). RT-qPCR in triplicate was done under the operation of Choromo 4 Bio-Rad machine (USA) and U6-snRNA was used as calibrator gene.

### Ethical consideration

A written informed consent form was obtained from all studied subjects. The study protocol were approved by the institutional review board of Royan Institute (Project ID. No. 94000096).

### Statistical analysis

The mean Cq of *hsa-miR-27a-3p* between OA and NOA was compared using *t* test. In addition, the GraphPad Prism 7.05 software (USA) was used and p-values < 0.05 were considered as significant.

## 3. Results

### Bioinformatics exploration

Delving in various databases, the existing information was screened deeply and *hsa-miR-27a-3p* was selected. The *hsa-miR-27a-3p* showed the highest frequency in different databases, the highest score of potentiality of targeting *KDM3A* transcript, and conserved seed sequences among all vertebrates. In vitro studies were also conducted on *hsa-miR-27a-3p* miRNA to affirm the rate of *KDM3A* targeting.

### RNA extraction and RT-qPCR analysis

**Figure 1:**
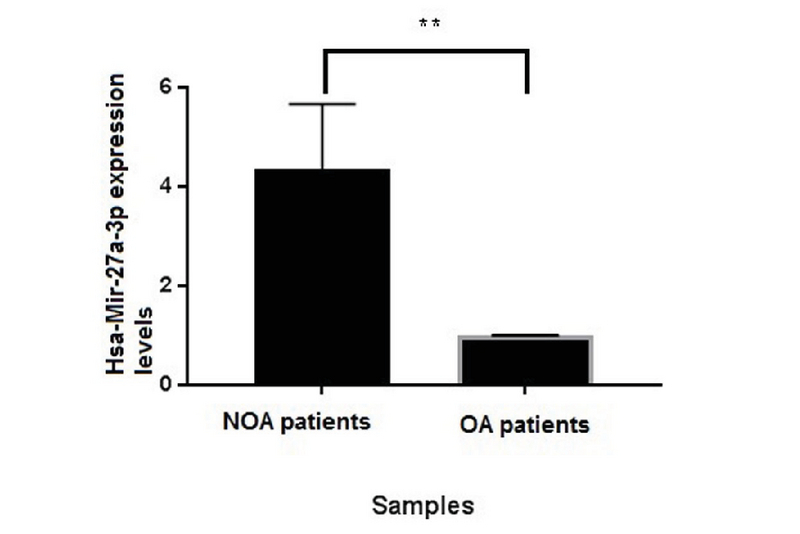
Hsa-miR-27a-3p mean expression level between NOA vs OA.

The concentration of the purified RNA was determined by reading the RNA absorbance at wavelengths of 260, 280, and 230 nm. The absorptionratios of 260/280 and 260/230 were between 1/8 and 2. For quality evaluation of the extracted RNA, electrophoresis gel was run. In addition, to find the *hsa-miR-27a-3p* expression between NOA and OA men, the RT-qPCR technique was performed. Primer specificity was evaluated by melt curve analysis which has shown the best specificity for the primers that has been depicted by a single pick. The *hsa-miR-27a-3p* expression was normalized by U6 snRNA internal control and the expression levels of NOA and OA were compared. P = 0.019 was calculated through statistical analysis of the RT-qPCR data via *t* test which shows the meaningful overexpression of *hsa-miR-27a-3p* in NOA men. The *hsa-miR-27a-3p* miRNA mean expression level was drawn via Graph Pad Prism 7.05 software (Figure 1).

## 4. Discussion

In this study, it was found that *hsa-miR-27a-3p* overexpress in NOA men than OA men and this miRNA can potentially match with the 3'UTR of *KDM3A* transcript and prevent its translation and lead to infertility in NOA men.

Finding the predictive markers for sperm retrieval is very important because they could be used as diagnostic agents for infertile couples. To achieve this goal, many investigations have been done to find a valuable marker for sperm retrieval (16-18). The previous study conducted by Tournaye and colleagues that focused on the non-genetic markers such as serum FSH level, semen analysis, and testis dimensions found that these non-genetic factors do not have enough ability to predict sperm retrieval (19). A similar study to find sperm retrieval-predicting markers was conducted on the blood samples of 78 NOA, 15 OA, and 10 fertile men measuring the FSH and Inhibin B serum factors (20). The study proposed that NOA men have a significantly lower inhibin B protein and high FSH level than OA men, and that because of this, inhibin B may be used as an accurate and reliable marker for evaluating sperm retrieval (20). Because the expression of inhibin B depends on FSH hormone and regarding the change of male hormones level by ageing as the change of FSH level, inhibin B cannot be used as an accurate marker for fertility evaluation of infertile men (21).

Moreover, the first study to find the genetic markers in azoospermia men was conducted by Lee and colleagues. They investigated the *DAZ* and *DAZL1* genes expression in Korean azoospermic men via RT-qPCR method. They assessed the genes expression of 43 infertile men, including 20 SCOS, 6 MA, 6 natural spermatogenesis, and 6 hypospermatogenesis men. The microscopic study of seminiferous tubules revealed that spermatogenetic cells were observed only in 30% of SCOC men. While *DAZ* and *DAZL1* genes expression were reported in 13 men with spermatozoa in microscopic cuts, no expression of the mentioned genes was seen in 14 men with no spermatogenic cells (22). The first case of RT-qPCR method usage in gene expression as a genetic marker for sperm retrieval evaluation was reported in 2002. This study conduct on 23 idiopathic azoospermia men and 5 fertile men as the control group for analyzing the *DAZ*, *RBMY1*, *USP9Y*, *protamine-2*, *SRY*, and *actin* gene expression. This study showed that the spermatogenesis genes expression of azoospermic patients are different from that of fertile men (23).

In a study performed on mouse models, it was found that partial deletion of *KDM3A* gene leads to a significant decrease in the number of sperms in mice (6). In subsequent studies, it was indicated that *KDM3A* controls the two downstream genes of *PRM1* and *TNP1*, and the lack of *KDM3A* expression causes downregulation of the *PRM1* and *TNP1* expressions (6-8). Also, regarding the function of demethylase of the *KDM3A* gene on the lysine-9 domain of H3, it was found that methylation of the promoter of the two genes of *TNP1* and *PRM1* was also significantly increased (6, 7). The results of that research showed that the expression of all the studied genes including *KDM3A*, *TNP1*, and *PRM1* in all histological groups of azoospermia men with the tissue pattern of SCOC suggested a significant difference in the expression of hypospermatogenesis (7).

One of the biomarkers that has attracted the attention of many researchers is non-coding RNA including miRNAs. The abnormal expression of miRNAs are related to many diseases and abnormalities (11). In a study that conducted on 200 azoospermia men its was showen that miR-141, miR-429, and miR-7-1-3p upregulated in NOA men (24). Likewise, another study performed to find altered miRNA expression profile in infertile male and found 54 miRNAs upregulated and 27 miRNAs downregulated in asthenozoospermic men (25). Microarray analysis indicated 93 miRNAs and 4,172 mRNAs were differentially expressed in the NOA and normozoospermic OA men, respectively (26).

In 2018, a study was performed on the miRNA27a and its association with pancreatitis cancer (27). In this study, While miRNA27a mimic was applied to overexpress the miRNA27a, an anti miR27a was applied to lower express the same (27). The results demonstrated that the overexpression of *hsa-miR-27a-3p* leads to cell growth and apoptosis prevention, while the lower expression of it leads to cell growth prevention, leading the cell to apoptosis (27). In a study on cervical cancer, it was found that the papillomavirus blocks mir-27a transcription in its host (28). This study was done by Luciferase method, and the results showed that mir-27a directly targets 3'UTR of beta growth receptor agent (TGF-βRI) transcript and it causes a decrease in signaling of TGF-β (28). Consequently, it leads to inhibition of the signaling path of TGF-β, which acts as a tumor suppressor that causes cancer in uterine cells and especially in adenocarcinoma (28). In a study wich performed on pancreatitis cancer, it was found that in patients with this cancer, mir-27a overexpressed target 3'UTR Sprouty2 mRNA (29). Further, a low expression of Sprouty2 mRNA leads to growth, colony formation, and migration of pancreatitis cancer cells (29). In another study that was done on pulmonary cancer cells in mouse, it was found that the increased expression of mir-27a leads to cell migration, invasion, and angiogenesis by targeting the downstream genes, while decreasing the expression of this miRNA has a quite opposite effect (30).

According to the former researches on the *hsa-miR-27a-3p* and its association with human diseases and abnormalities (27-30), and also by using in silico and in vitro studies, it supports the idea that *hsa-miR-27a-3p* overexpressed in NOA men. The former investigation illustrated that *KDM3A* transcript significantly decreased in NOA men (6-8). Based on in silico studies, *hsa-miR-27a-3p* has the potential ability to target *KDM3A* transcript in testicular tissue by attaching on 3'UTR of *KDM3A* mRNA and hinder its translation. Consequently, downstream genes which are controlled by *KDM3A* such as *PRM1* and *TNP1* are not expressed and all these together drive to male infertility in NOA men. The discovery of new miRNAs which are associated with the specific human disease or abnormality can finally lead to a change in the attitude of treatment methods toward infertile couples and also regarding the possibility of using miRNA-based drugs.

## 5. Conclusion

In this study, it was showed that *hsa-miR-27a-3p* overexpressed in NOA testicular tissue in comparison to the OA men. In silico investigations propose that the most probable target for *hsa-miR-27a-3p* in testicular tissue is *KDM3A* transcript. Also, in silico studies suggest that *hsa-miR-27a-3p* could complementarily match to the 3'UTR of *KDM3A* transcription and hinder its translation. As the product of *KDM3A* regulates its downstream genes such as *TNP1* and *PRM1* expression, in the lack of *KDM3A* product, these genes are staying off (7). Consequently, the lack of *TNP1* and *PRM1* genes expression leads to the loss of the required condensation of chromosomes for proper packing in the sperm head, and it is considered as one of the causes of infertility in NOA men (7).

##  Conflict of Interest

None of the authors have any conflicts of interest to declare and all authors support submission to this journal.

## References

[1] Fernández Pelegrina R, Kessler AG, Rawlins RG. Modern approaches to the treatment of human infertility through assisted reproduction. *P R Health Sci J* 1991; 10: 75–81. 1946922

[2] Cooper TG, Noonan E, Von Eckardstein S, Auger J, Baker HW, Behre HM, et al. World Health Organization reference values for human semen characteristics. *Hum Reprod Update* 2010; 16: 231–245. 10.1093/humupd/dmp048 19934213

[3] Irvine DS. Epidemiology and aetiology of male infertility. *Hum Reprod* 1998; 13 (Suppl.): 33–44. 10.1093/humrep/13.suppl_1.33 9663768

[4] Aziz N. The importance of semen analysis in the context of azoospermia. *Clinics* 2013; 68 (Suppl.): 35–38. 10.6061/clinics/2013(Sup01)05 PMC3583176 23503953

[5] Jarow JP, Espeland MA, Lipshultz LI. Evaluation of the azoospermic patient. *J Urol* 1989; 142: 62–65. 10.1016/s0022-5347(17)38662-7 2499695

[6] Okada Y, Tateishi K, Zhang Y. Histone demethylase JHDM${}_{2}$A is involved in male infertility and obesity. *J Androl *2010; 31: 75–78. 10.2164/jandrol.109.008052 PMC4096721 19875498

[7] Javadirad SM, Hojati Z, Ghaedi K, Nasr-Esfahani MH. Expression ratio of histone demethylase KDM 3A to protamine-1 mRNA is predictive of successful testicular sperm extraction in men with obstructive and non-obstructive azoospermia. *Andrology* 2016; 4: 492–499. 10.1111/andr.12164 27027467

[8] Okada Y, Scott G, Ray MK, Mishina Y, Zhang Y. Histone demethylase JHDM${}_{2}$A is critical for Tnp1 and Prm1 transcription and spermatogenesis. *Nature* 2007; 450: 119–123. 10.1038/nature06236 17943087

[9] Zamudio NM, Chong S, O'Bryan MK. Epigenetic regulation in male germ cells. *Reproduction* 2008; 136: 131–146. 10.1530/REP-07-0576 18515312

[10] Krol J, Loedige I, Filipowicz W. The widespread regulation of microRNA biogenesis, function and decay. *Nat Rev Genet *2010; 11: 597–610. 10.1038/nrg2843 20661255

[11] Heneghan HM, Miller N, Kerin MJ. MiRNAs as biomarkers and therapeutic targets in cancer. *Curr Opin Pharmacol* 2010; 10: 543–550. 10.1016/j.coph.2010.05.010 20541466

[12] Mattick JS. Non-coding RNAs: the architects of eukaryotic complexity. *EMBO Rep* 2001; 2: 986–991. 10.1093/embo-reports/kve230 PMC1084129 11713189

[13] Charan J, Biswas T. How to calculate sample size for different study designs in medical research? *Indian J Psychol Med *2013; 35: 121–126. 10.4103/0253-7176.116232 PMC3775042 24049221

[14] Bonaparte E, Moretti M, Colpi GM, Nerva F, Contalbi G, Vaccalluzzo L, et al. ESX1 gene expression as a robust marker of residual spermatogenesis in azoospermic men. *Hum Reprod* 2010; 25: 1398–1403. 10.1093/humrep/deq074 20356899

[15] Pansa A, Sirchia SM, Melis S, Giacchetta D, Castiglioni M, Colapietro P, et al. ESX1 mRNA expression in seminal fluid is an indicator of residual spermatogenesis in non-obstructive azoospermic men. *Hum Reprod* 2014; 29: 2620–2627. 10.1093/humrep/deu261 25316452

[16] Schlegel PN, Li PS. Microdissection TESE: sperm retrieval in non-obstructive azoopsermia. *Hum Reprod Update* 1998; 4: 439. 10.1093/humupd/4.4.439 9825858

[17] Song GJ, Lee H, Park Y, Lee HJ, Lee YS, Seo JT, et al. Expression pattern of germ cell-specific genes in the testis of patients with nonobstructive azoospermia: usefulness as a molecular marker to predict the presence of testicular sperm. *Fertil Steril *2000; 73: 1104–1108. 10.1016/s0015-0282(00)00520-3 10856465

[18] Seo JT, Ko WJ. Predictive factors of successful testicular sperm recovery in non-obstructive azoospermia patients. *Int J Androl* 2001; 24: 306–310. 10.1046/j.1365-2605.2001.00307.x 11554989

[19] Tournaye H, Verheyen G, Nagy P, Ubaldi F, Goossens A, Silber S, et al. Are there any predictive factors for successful testicular sperm recovery in azoospermic patients? *Hum Reprod *1997; 12: 80–86. 10.1093/humrep/12.1.80 9043908

[20] Brugo-Olmedo S, De Vincentiis S, Calamera JC, Urrutia F, Nodar F, Acosta AA. Serum inhibin B may be a reliable marker of the presence of testicular spermatozoa in patients with nonobstructive azoospermia. *Fertil Steril *2001; 76: 1124–1129. 10.1016/s0015-0282(01)02866-7 11730738

[21] Meachem SJ, Nieschlag E, Simoni M. Inhibin B in male reproduction: pathophysiology and clinical relevance. *Eur J Endocrinol* 2001; 145: 561–571. 10.1530/eje.0.1450561 11720872

[22] Lee JH, Lee DR, Yoon SJ, Chai YG, Roh SI, Yoon HS. Expression of DAZ (deleted in azoospermia), DAZL1 (DAZ-like) and protamine-2 in testis and its application for diagnosis of spermatogenesis in non-obstructive azoospermia. *Mol Hum Reprod* 1998; 4: 827–834. 10.1093/molehr/4.9.827 9783841

[23] Kleiman SE, Yogev L, Hauser R, Botchan A, Maymon BS, Paz G, et al. Expression profile of AZF genes in testicular biopsies of azoospermic men. *Hum Reprod* 2007; 22: 151–158. 10.1093/humrep/del341 16936303

[24] Wu W, Qin Y, Li Z, Dong J, Dai J, Lu C, et al. Genome-wide microRNA expression profiling in idiopathic non-obstructive azoospermia: significant up-regulation of miR-141, miR-429 and miR-7-1-3p. *Hum Reprod* 2013; 28: 1827–1836. 10.1093/humrep/det099 23559187

[25] Abu-Halima M, Hammadeh M, Schmitt J, Leidinger P, Keller A, Meese E, et al. Altered microRNA expression profiles of human spermatozoa in patients with different spermatogenic impairments. *Fertil Steril *2013; 99: 1249–1255. e16. 10.1016/j.fertnstert.2012.11.054 23312218

[26] Zhuang X, Li Z, Lin H, Gu L, Lin Q, Lu Z, et al. Integrated miRNA and mRNA expression profiling to identify mRNA targets of dysregulated miRNAs in non-obstructive azoospermia. *Sci Rep* 2015; 5: 7922. 10.1038/srep07922 PMC4310093 25628250

[27] Cui Z, Liu G, Kong D. miRNA-27a promotes the proliferation and inhibits apoptosis of human pancreatic cancer cells by Wnt/β-catenin pathway. *Oncol Rep* 2018; 39: 755–763. 10.3892/or.2017.6120 29207155

[28] Fang F, Huang B, Sun S, Xiao M, Guo J, Yi X, et al. miR-27a inhibits cervical adenocarcinoma progression by downregulating the TGF-βRI signaling pathway. *Cell Death Dis* 2018; 9: 395–408. 10.1038/s41419-018-0431-2 PMC5847584 29531222

[29] Gao W, Hong Z, Huang H, Zhu A, Lin S, Cheng C, et al. miR-27a in serum acts as biomarker for prostate cancer detection and promotes cell proliferation by targeting Sprouty2. *Oncol Lett* 2018; 16: 5291–5298. 10.3892/ol.2018.9274 PMC6144816 30250598

[30] Wang YL, Gong WG, Yuan QL. Effects of miR-27a upregulation on thyroid cancer cells migration, invasion, and angiogenesis. *Genet Mol Res* 2016; 15: 1–10. 10.4238/gmr15049070 28002594

